# A new recumbirostran ‘microsaur’ from the lower Permian Bromacker locality, Thuringia, Germany, and its fossorial adaptations

**DOI:** 10.1038/s41598-023-46581-3

**Published:** 2024-02-20

**Authors:** Mark J. MacDougall, Andréas Jannel, Amy C. Henrici, David S. Berman, Stuart S. Sumida, Thomas Martens, Nadia B. Fröbisch, Jörg Fröbisch

**Affiliations:** 1 Museum für Naturkunde Leibniz-Institut für Evolutions- und Biodiversitätsforschung, Invalidenstraße 43, 10115 Berlin, Germany; 2 Carnegie Museum of Natural History, Pittsburgh, PA USA; 3 California State University, San Bernardino, CA USA; 4 99869 Drei Gleichen, Germany; 5 Humboldt-Universität zu Berlin, Invalidenstraße 42, 10115 Berlin, Germany

**Keywords:** Zoology, Evolution, Palaeontology, Phylogenetics, Taxonomy

## Abstract

Several recumbirostran ‘microsaurs’ are known from early Permian sites across Germany, including the Tambach Formation in Thuringia, central Germany. The only ‘microsaur’ thus far described from the Tambach Formation was the ostodolepid recumbirostran *Tambaroter carrolli*. However, there is also the documented presence of an undescribed recumbirostran ‘microsaur’ at the well-known Bromacker locality. The Bromacker locality is highly recognized and best known for its very diverse and extremely well-preserved terrestrial tetrapod assemblage combined with the co-occurrence of an exceptional vertebrate ichnofossil record. Here we describe a second new recumbirostran taxon from the Tambach Formation, which is also the first from the Bromacker locality itself. Phylogenetic analysis indicates that the new taxon, *Bromerpeton subcolossus* gen. et sp. nov., is a brachystelechid recumbirostran, a group also known elsewhere in Germany. The following features differentiate *Bromerpeton* from the other members of the clade: the presence of 13 maxillary teeth, narrow postorbitals that do not substantially contribute to the postorbital region of the skull, the frontal does not contribute to the orbital margin, and the presence of five manual digits. This new recumbirostran ‘microsaur’ further adds to the unique ecosystem that is preserved at the Bromacker locality, granting us a better understanding of what was living underfoot the larger more well-known animals at the locality. Likewise, it expands our understanding of the evolution of recumbirostran ‘microsaurs’, especially with regards to digit and limb reduction within the clade.

## Introduction

The Bromacker locality, located in Thuringia, Germany, has been interpreted as one of only a few known inland terrestrial assemblages of the early Permian^1–5^. Based on biostratigraphic data the Tambach Formation is currently considered to be Artinskian in age^6^. Unfortunately, a more accurate absolute age cannot be determined due to the absence of volcanic rocks in the Tambach Formation, but the underlying Rotterode Formation has as an absolute age of approximately 296 Ma^7^, which limits the maximum possible age for the Tambach Formation to potentially early Sakmarian/late Asselian, as has been suggested recently^8,9^. The Bromacker locality preserves a unique fauna dominated by the herbivorous diadectids^2–4,10^. In addition to the substantial diadectid component of the fauna, various terrestrial anamniote tetrapods^1,4,11–13^, reptiles^4,14,15^, and synapsids^4,16–18^ are also known from the Bromacker locality, indicating a diverse assemblage of early tetrapods.

Among the numerous taxa currently known from the Tambach Formation is a single ‘microsaur’, the ostodolepid recumbirostran *Tambaroter carrolli*, though not discovered at the Bromacker quarry itself, but rather from an excavation at a construction site in the nearby town of Tambach-Dietharz^19^. Currently it is unknown if this site falls within the same fossiliferous stratigraphic horizon of the Tambach Formation as the Bromacker quarry, an issue that will be addressed by ongoing geological studies of the Tambach Basin. Aside from *Tambaroter*, several other ‘microsaurs’ are known form the early Permian of Germany, with *Altenglanerpeton schroederi* and four species of the brachystelechid *Batropetes* from the Saar-Nahe Basin of southwestern Germany^20,21^, and the enigmatic *Saxonerpeton geinitzi* from the Döhlen Basin in Saxony^22^. Eberth et al. [^4^] previously mentioned but did not describe a possible tuditanomorph ‘microsaur’ from the Bromacker locality.

Here, we describe this previously mentioned ‘microsaur’ specimen from the Bromacker locality, *Bromerpeton subcolossus* gen. et sp. nov., which represents a new recumbirostran from the Bromacker locality. An updated phylogenetic analysis is conducted, indicating that the new taxon belongs to the recumbirostran clade Brachystelechidae. We discuss the importance of studying the smaller and often less complete taxa from the locality, as they greatly increase our understanding of the ecosystem that was present at Bromacker during the early Permian.

*Institutional abbreviations:* MNG—Museum der Natur, Stiftung Schloss Friedenstein, Gotha, Germany.

## Material and methods

### Fossil material

**Figure 1:**
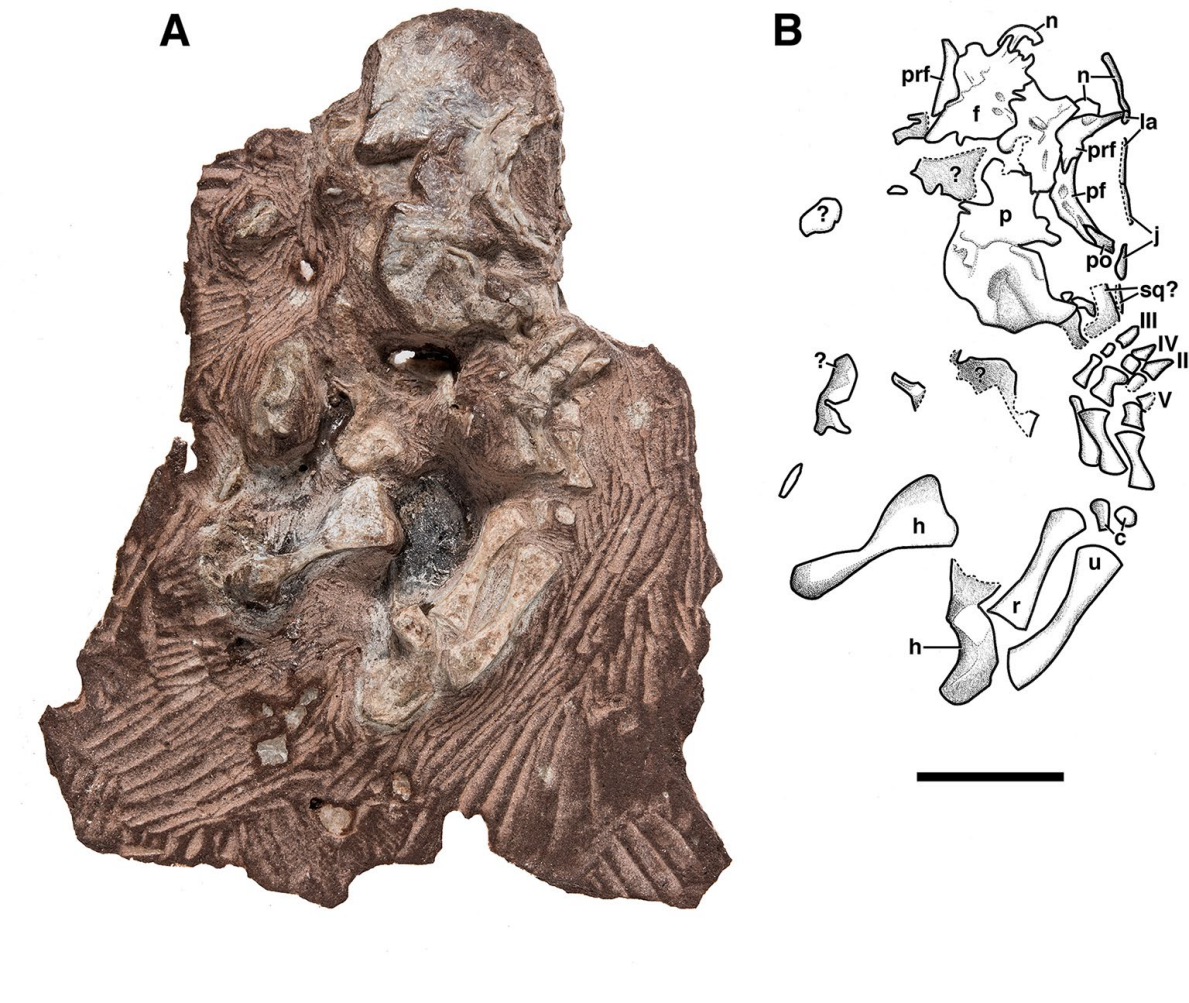
Holotype of *Bromerpeton subcolossus*, MNG 16545, photograph and interpretive drawing in dorsal view. Abbreviations: **c**, carpal; **f**, frontal; **h**, humerus; **j**, jugal; **la**, lacrimal; **n**, nasal; **p**, parietal; **pf**, postfrontal; **po**, postorbital; **prf**, prefrontal; **r**, radius; **sq?**, possible squamosal; **u**, ulna; **II**–**IV**, digits two to four (the first digit is not visible externally); **?**, unidentifiable fragment. Scale bar equals 10 mm.

**Figure 2:**
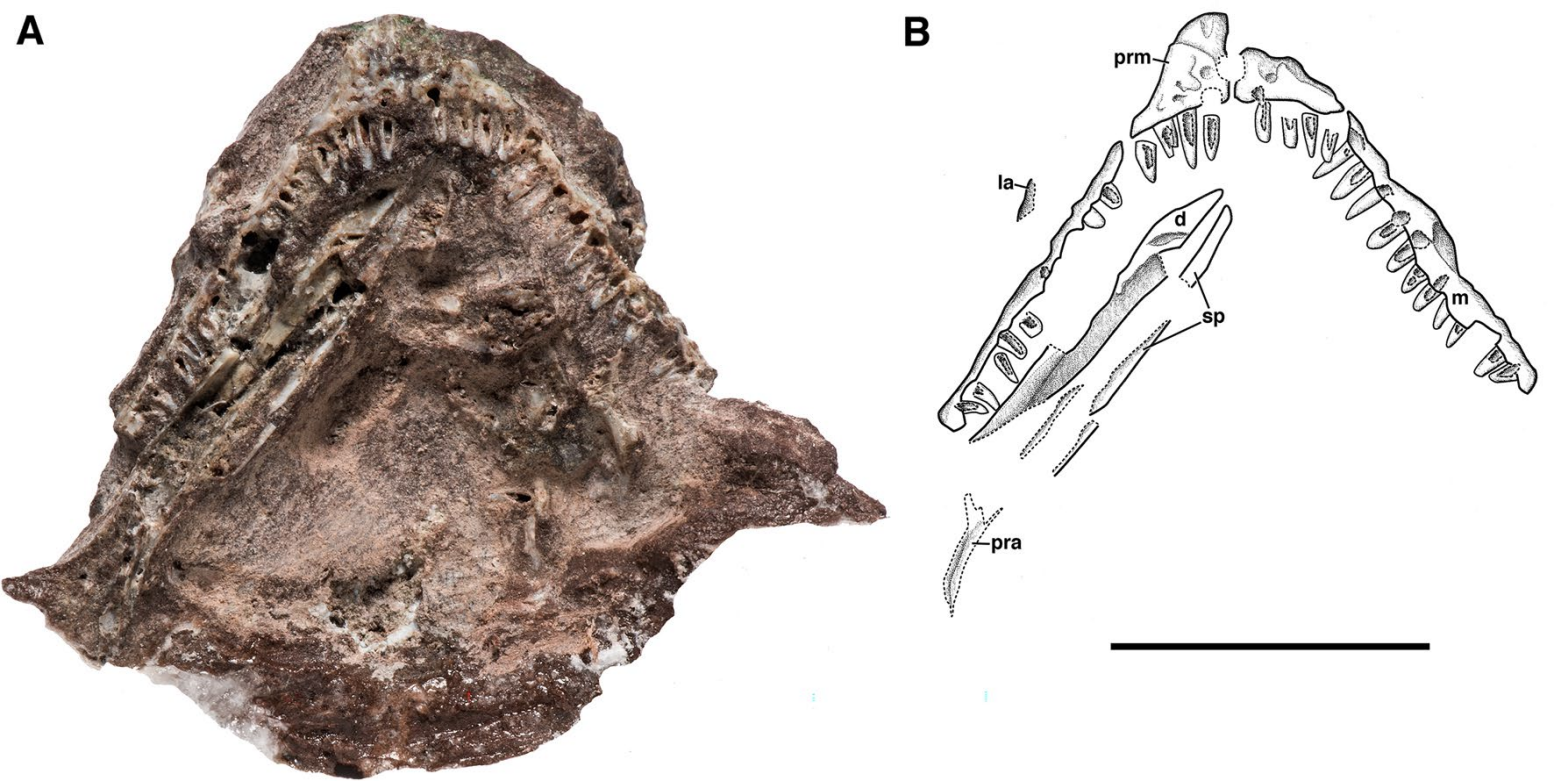
Holotype of *Bromerpeton subcolossus*, MNG 16545, photograph and interpretive drawing in ventral view. Abbreviations: **d**, dentary; **la**, lacrimal; **m**, maxilla; **pra**, prearticular; **prm**, premaxilla; **sp**, splenial; **?**, unidentifiable fragment. Scale bar equals 10 mm.

The holotype and only currently known specimen of *Bromerpeton subcolossus*, MNG 16545, was discovered during the field season in 1994 and was subsequently prepared at the Carnegie Museum, Pittsburgh. Further preparation of the skull was undertaken at the Museum für Naturkunde Berlin. The specimen consists of a partial skull and anterior elements of the postcranium (Figs. 1, 2). The skull is incomplete with several elements of the skull roof and mandible being either absent, damaged, or preserved as impression. The entire skull is dorsoventrally compressed due to taphonomic factors, obscuring and distorting some of the preserved elements. Many postcranial elements are not preserved, notably most of the axial skeleton, the pelvic girdle, and the hindlimbs.

### Computed tomography scanning and segmentation

MNG 16545 was scanned using the x-ray computed tomography equipment (Phoenixǀx-ray Nanotomǀs) at the Museum für Naturkunde in Berlin. Scan parameters were set to 110 kV voltage and 85µA current with 1440 images /360° at an exposure time of 1000 ms/image and an effective voxel size of 0.0305 mm, resulting in a magnification rate of 3.857. Cone beam reconstruction was performed using datosǀx-ray sensing 4 Inspection Technologies GmbH (phoenixǀx-ray) with a correction value of 1.845. Elements were visualized and digitally segmented in VGStudio Max 3.4.3. The 3D models resulting from the segmentation were outputted to Wavefront Object file format (*.obj) and imported into Autodesk Maya 2019 (www.autodesk.com), where the full set of osteological elements were positioned into a more natural position. Raw CT data used in this study can be accessed at MorphoSource, https://www.morphosource.org/projects/000449864.

### Phylogenetic analysis

The new specimen was coded into the character-taxon matrix of Mann et al.^23^, the most current matrix available for investigating recumbirostran phylogeny. The matrix was further updated with the addition of *Tambaroter carrolli*, another recrumbirostran from the Tambach Formation, and updated character codings for *Nannaroter mckinziei* were incorporated based on MacDougall et al.^24^. *Tambaroter* was coded using the holotype, and only known specimen, MNG 14708. The phylogenetic analysis was performed in PAUP 4.0a169^25^ with maximum parsimony set as the optimality criterion, all branch lengths of less than zero were set to collapse, and a heuristic search with 1000 random additional replicates and tree bisection and reconnection (TBR) branch swapping was used to search for trees. *Eusthenopteron* was designated as the outgroup taxon for the analysis. The character codings for both MNG 16545 and MNG 14708 can be found in the data matrix included in the supplementary information. Both a bootstrap analysis, using fast stepwise addition (1000 replicates), and a Bremer decay analysis were conducted to determine the support values for the recovered nodes.

## Systematic Palaeontology

TETRAPODA Jaekel, 1909^26^

RECUMBIROSTRA Anderson, 2007^27^

BRACHYSTELECHIDAE Carroll and Gaskill, 1978^22^

*BROMERPETON SUBCOLOSSUS* gen. et sp. nov.

(Figs. 1, 2 and 3).

**Figure 3:**
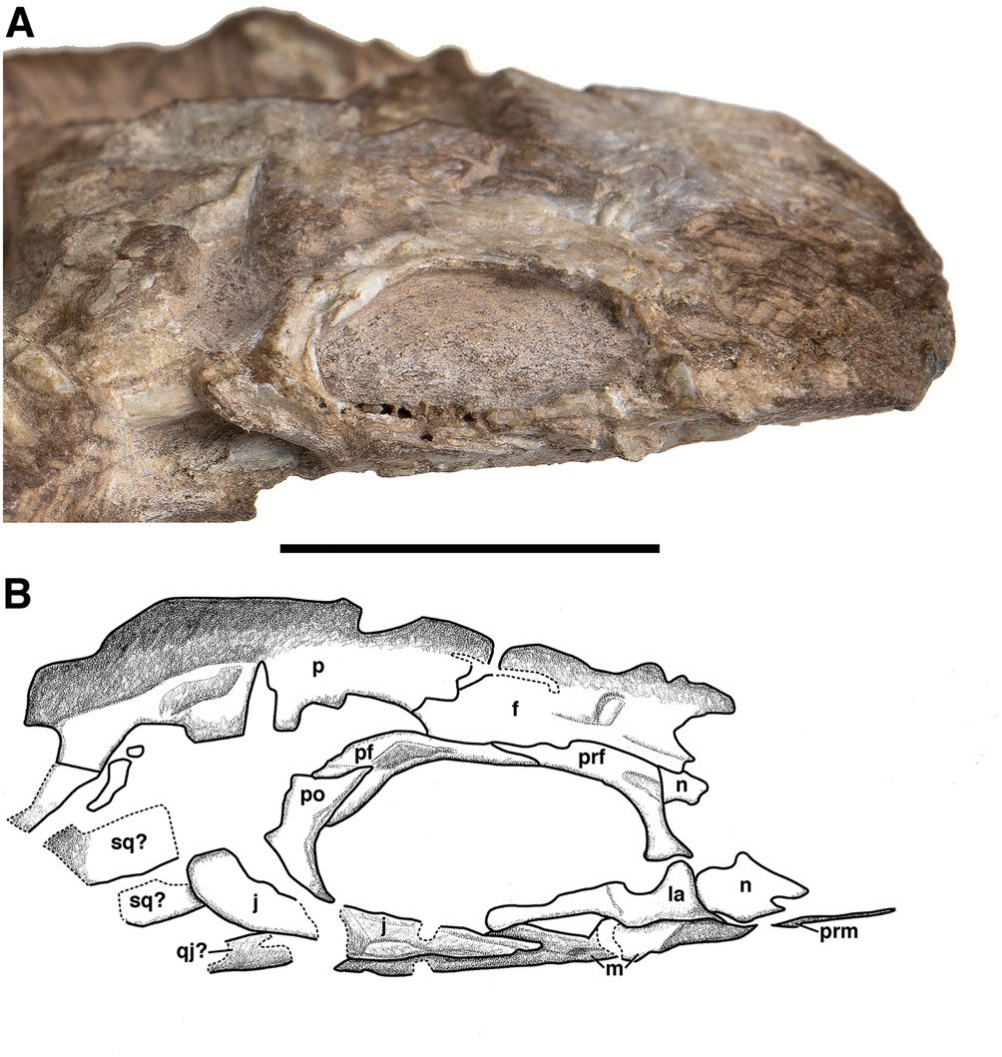
Holotype of *Bromerpeton subcolossus*, MNG 16545, photograph and interpretive drawing in right lateral view. Abbreviations: **f**, frontal; **j**, jugal; **la**, lacrimal; **m**, maxilla; **n**, nasal; **p**, parietal; **pf**, postfrontal; **po**, postorbital; **prf**, prefrontal **prm**, premaxilla; **qj?**, possible quadratojugal; **sp**, splenial; **sq?**, possible squamosal. Scale bar equals 10 mm.

[urn:lsid:zoobank.org:act:78B072B4-27B3-42B3-8E67-7D349A4F719A (genus)]

[urn:lsid:zoobank.org:act:769BA815-ADFA-4C05-9769-F044C37392AA (species)]

*Holotype*: MNG 16545, a partial skull and mandible with left humerus and largely complete right forelimb.

*Diagnosis:* Brachystelechid recumbirostran diagnosed by the following characters: 13 maxillary teeth, frontals do not contribute to the orbital margin, narrow postorbitals do not contribute substantially to the postorbital region of the skull, and presence of five manual digits. In all other brachystelechids the prefrontal and postfrontal do not meet, which allows the frontal to contribute to the dorsal edge of the orbital margin. Shares with *Diabloroter*, but differs from *Carrolla* and *Batropetes* in exhibiting a homodont dentition of small monocuspid teeth.

*Etymology*: Genus name derives from Bromacker (which translates to Brom’s Field in English) the name of the locality that the specimen was discovered at, and *erpeton* is a common epithet for small early tetrapods, meaning creeper in Greek. Species name derives from the Latin words for below and colossus, referring to the small size of this species relative to the abundant and much larger vertebrates of the fauna.

*Locality and horizon:* Bromacker locality, quarry near the town of Tambach-Dietharz, Thuringia, Germany. Located in the Upper Beds of the Lower Permian Tambach Formation^4^, Artinskian in age based on biostratigraphy^6^, but potentially could be as old as early Sakmarian/late Asselian^7–9^.

## Nomenclatural acts

The electronic version of this article conforms to the requirements of the International Code of Zoological Nomenclature (ICZN), and the new names contained herein are available under that Code from the electronic version of this article. This published work and the nomenclatural acts it contains have been registered in ZooBank, the proposed online registration system for the ICZN. The ZooBank Life Science Identifiers (LSIDs) can be resolved and the associated information viewed through any standard web browser by appending the LSID to the prefix http://zoobank.org/. The LSIDs for this publication are: urn:lsid:zoobank.org:pub:09C76579-1F21-4E7A-8C85-CF7FFE6FF6A7 (article); urn:lsid:zoobank.org:act:78B072B4-27B3-42B3-8E67-7D349A4F719A (genus); urn:lsid:zoobank.org:act:769BA815-ADFA-4C05-9769-F044C37392AA (species).

## Description

### General

The skull of MNG 16545 (Figs. 1, 2) is dorsoventrally compressed, causing the lateral and anterior surfaces of the maxilla and premaxilla to be exposed in ventral aspect and damage to other portions of the skull, especially the cheek region. Portions of the specimen are weathered, most likely due to exposure to mud-laden ground water that flows along joints in the quarry subsurface. This caused possible loss of some skull roofing bones (e.g. nasals) and hollowing out other bones and teeth. Furthermore, the quality of the CT scans of MNG 16545 were highly variable depending on the region, and they did not offer satisfactory resolution for most of the skull region. This is common among the Bromacker fossils due to the variable density of the matrix (MM pers. obs.) In combination, these factors make it difficult to impossible to identify some of the bones.

### Skull roof

The skull is small, being roughly 20 mm in length, but it appears to be relatively wide posteriorly at the level of the parietals, which are approximately 16 mm in width, an estimation based on the widest part of the right parietal (8 mm). Very little sculpturing is present on the preserved elements of the skull roof. What is present consists mostly of faint ridges and a few small pits located on the premaxilla, frontal, prefrontal, and postfrontal.

The paired premaxillae are only visible in ventral view (Fig. 2) and due to dorsoventral compression of the skull they are pushed posteriorly, which has exaggerated the recumbent snout that characterizes many members of Recumbirostra^27^. A broad, slightly curved dorsal process is present that would have articulated with the nasal, which are represented by a few small fragments. It does not appear that there would have been any substantial dorsal exposure, because the premaxillae are not visible dorsally, as in *Quasicaecilia*^28^ and *Diabloroter*^29^. Each premaxilla bears four small, pointed, monocuspid teeth, which is less than in *Tambaroter*^19^, *Proxilodon*^30^, *Rhynchonkos*, *Aletrimyti*, and *Dvellacanus*^31^, all of which have five premaxillary teeth, but it is equivalent to what occurs in *Carrolla*^32^ and *Euryodus*^33^. This tooth number is also less than in the early recumbirostran *Steenerpeton,* which possesses 7 premaxillary teeth^34^, suggesting a reduction in tooth number over the course of recumbirostran evolution. There is also a small ventromedially placed foramen on each premaxilla.

Both maxillae are present, but are heavily damaged, with the most informative portions being the tooth bearing surface and associated marginal dentition (Fig. 2). As seen in ventral view (Fig. 2), the maxilla narrowly contacts the premaxilla near the mid length of the narial opening, and together they form its ventral margin. The tooth bearing surface of the left maxilla is the more complete, and exhibits 13 tooth positions, 11 of which bear small pointed teeth. Most of the teeth are damaged with the labial surface of the teeth being absent in places, resulting in the exposure of the pulp cavities in some of the teeth. However, the overall tooth shape is preserved, and it is apparent that they are identical in form to those of the premaxilla. The homodont dentition of small conical teeth is similar to that of other recumbirostrans such as the early-diverging *Steenerpeton*^34^, *Diabloroter*^29^ and *Odonterpeton*^23^, though it is quite distinct from the multicuspid teeth in brachystelechids such as *Batropetes* (Glienke, 2015) and *Carrolla*^32,35^, and the bulbous dentition of *Euryodus*^33^. The tooth bases also exhibit a loosely infolded dentine, similar to that in other early reptiles with shallowly implanted teeth^36^ and other recumbirostrans^24^.

Small fragmentary portions of the nasals are present, but little information can be obtained from them other than the contact of the fragmentary left nasal and frontal having a strongly interdigitating contact (Fig. 1).

The right lacrimal is also visible in lateral view (Fig. 3). It extends as a thin bar from a fragment of the nasal toward the anterior end of the jugal, near the mid length of the orbit, but does not contact it. This is likely due to the disarticulation of the jugal, and normally there would have been contact, as in *Carrolla*^32^ and *Batropetes*^21^. Anteriorly, the lacrimal expands dorsally to where it would have contacted the prefrontal, though both elements are slightly disarticulated and do not contact.

The jugal of MNG 16545 is damaged and disarticulated, being broken into two pieces, but in lateral view much of it is visible. It is a long element that extends from the postorbital to roughly the mid length of the orbit, narrowing anteriorly over its length until tapering to a point; overall it is much narrower than the dorsoventrally broad jugal of *Batropetes*^21^. Due to the disarticulation of the jugal, it is not in contact with the posterior end of the lacrimal, though it likely would have overlapped with the lacrimal normally. Ventral to the posterior end of the jugal is a fragmentary disarticulated element that may represent the quadratojugal.

Both prefrontals are present, with the right being more complete (Fig. 1). The latter extends from its posterior contact with the postfrontal and curves anteroventrally to reach the lacrimal. Anterodorsally, it contacts the fragmentary portion of the nasal. The prefrontal exhibits an overall narrow, curved shape, being broadest near its mid length and narrowing anteriorly and posteriorly, similar to the morphology observed in *Quasicaecilia*^28^.The posterior extension of the prefrontal has an interdigitating suture with the postfrontal, and together they form the dorsal margin of the orbit.

The right postfrontal is also present (Fig. 1), which is a narrow, curved element that extends posteroventrally from its anterior contact with the prefrontal and contributes to the posterodorsal margin of the orbit. Medially it contacts the frontal, and probably contacted the anterolateral edge of the parietal, but this cannot be positively determined due to the parietal being damaged. The posterior portion of the postfrontal curves slightly ventrally and contacts the postorbital.

The right postorbital is clearly visible in lateral view (Fig. 3) and is a lunate element with a curved anterior edge that contributes to the posterior margin of the orbit and narrows considerably toward both its dorsal and ventral ends. It is smaller than the large posteroventrally extending postorbitals of *Carrolla*^32^, *Batropetes*^21^, and *Diabloroter*^29^, though it may be missing part of its posterior extent. Dorsally, it contacts the postfrontal and ventrally it would have contacted the jugal, though the two elements are separated from one another in MNG 16545 due to damage and disarticulation of the jugal.

Both frontals are present, with the left frontal being more complete (Fig. 1). They are large anterodorsally expanded elements and extend from the anterior margin of the orbit to about its midpoint level. The more complete left frontal exhibits a small portion of an interdigitating contact with the fragmentary left nasal. The lateral extent of the frontal is excluded from the orbital margin by the prefrontal-postfrontal contact, a feature that is variable among recumbirostrans^19–21,30^, which could possibly be related to ontogeny. The suture between the frontals is deeply serrate and not interrupted by an interfrontal, which is present in some specimens of *Batropetes*^21^. Posteriorly, the right frontal contacts its associated parietal. Sculpturing in the form of sparse small pits and faint ridges is present on the frontals, although there is no organized sculpturing pattern on the frontals as observed in *Batropetes*^21^ and in *Diabloroter*^29^.

Only the right parietal is present, which is a broad, flat element that is by far the largest of the preserved skull roof (Fig. 1). Anteriorly the narrowest portion of the parietal contacts the frontal and broadens considerably posteriorly, as in *Carrolla*^32^, *Batropetes*^21^, *Quasicaecilia*^28^, *Diabloroter*^29^, *Joermungandr*^37^, and *Odonterpeton*^23^. Due to the incomplete nature of the skull its contacts with other elements of the skull roof cannot be determined. The medial margin near the anterior end of the right parietal is incised for its contribution to the anteriorly placed pineal foramen, which is large, but comparable in relative size to that of *Carrolla*^32^ and *Quasicaecilia*^28^.

Posterior to the right jugal and ventral to the right parietal are two fragments (Fig. 1, 3) that, based on their position, may represent the remains of the right squamosal.

### Mandible

**Figure 4:**
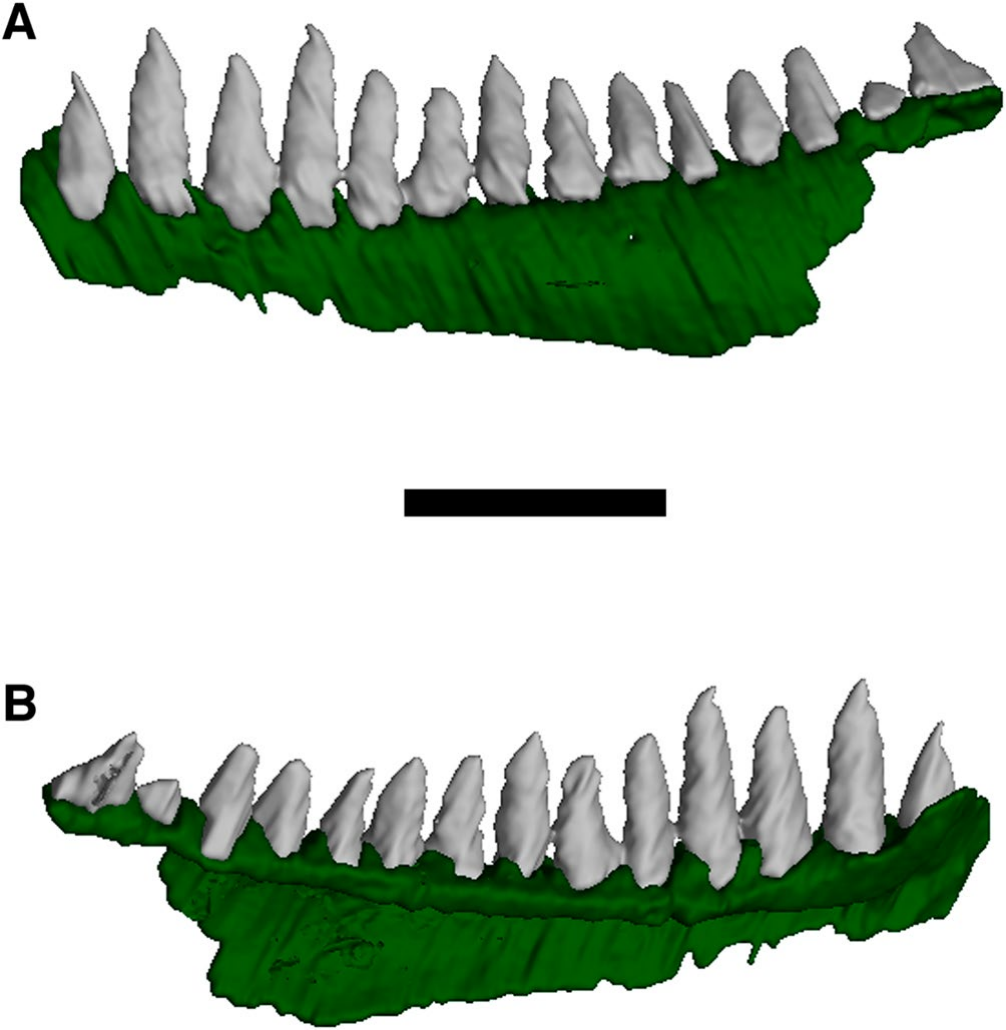
Computed tomography reconstruction of the left dentary of *Bromerpeton subcolossus*, MNG 16545, in (**A**), lateral, and (**B,**) medial views. Scale bar equals 3 mm.

A portion of the fragmentary right mandibular ramus is preserved adjacent to the right maxilla (Fig. 2). No informative details can be determined due to its position and poor preservation. However, CT scanning was used to identify a relatively intact left dentary and associated dentition (Fig. 4). The dentary is relatively short, and its dorsoventral height gradually increases posteriorly. It possesses 14 marginal teeth, all of which are very similar to those of the maxilla, being simple, pointed monocuspid teeth that are largely homodont. *Diabloroter*, with its similar dentition, possesses at least 18 dentary teeth^29^. In contrast other brachystelechids in which the dentary is known have less teeth, with eight tricuspid teeth in *Batropetes*^21^ and seven tricuspid teeth in *Carrolla*^32,35^.

### Postcranium

**Figure 5:**
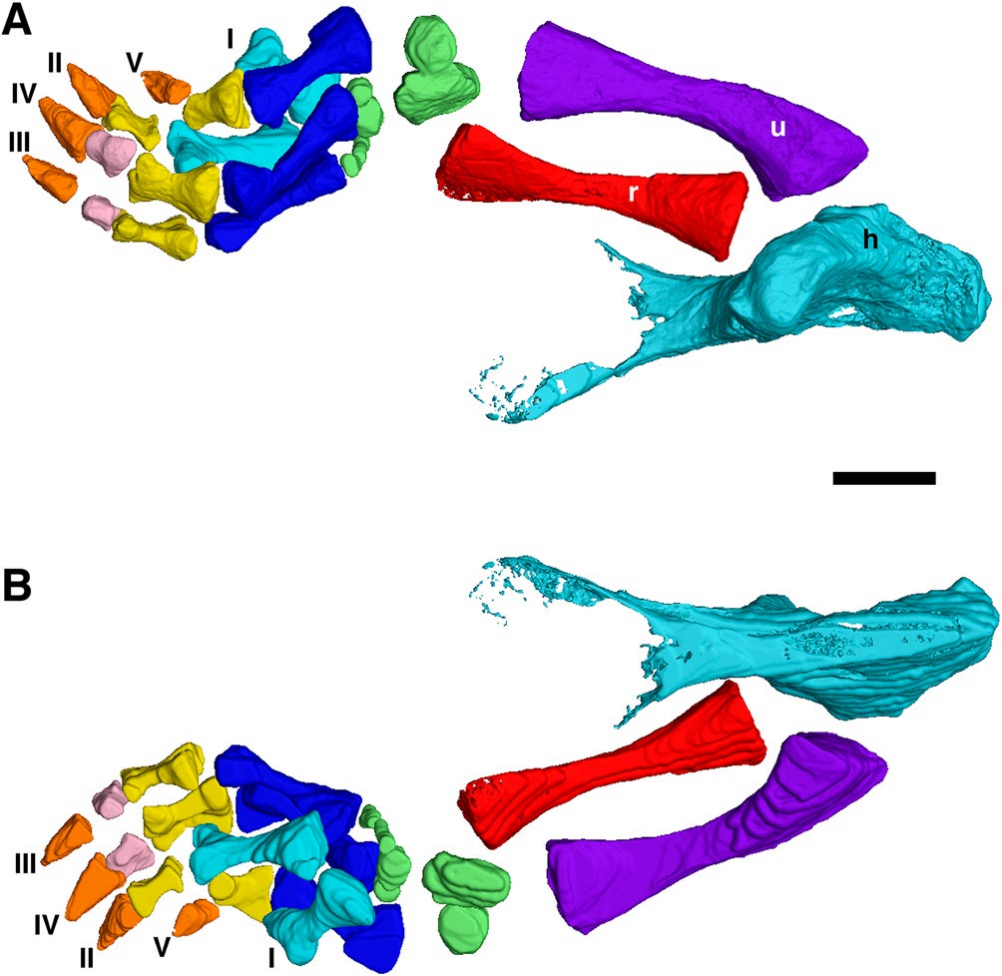
Computed tomography reconstruction of the right forelimb of *Bromerpeton subcolossus*, MNG 16545, in (**A**), dorsal, and (**B**), ventral views. Metacarpals not superficially exposed are indicated in a light shade of blue to distinguish them from overlying exposed metacarpals. Abbreviations: **h**, humerus; **r**, radius; **u**, ulna; **I**–**V**, digits 1 to 5. Scale bar equals 3 mm.

**Figure 6:**
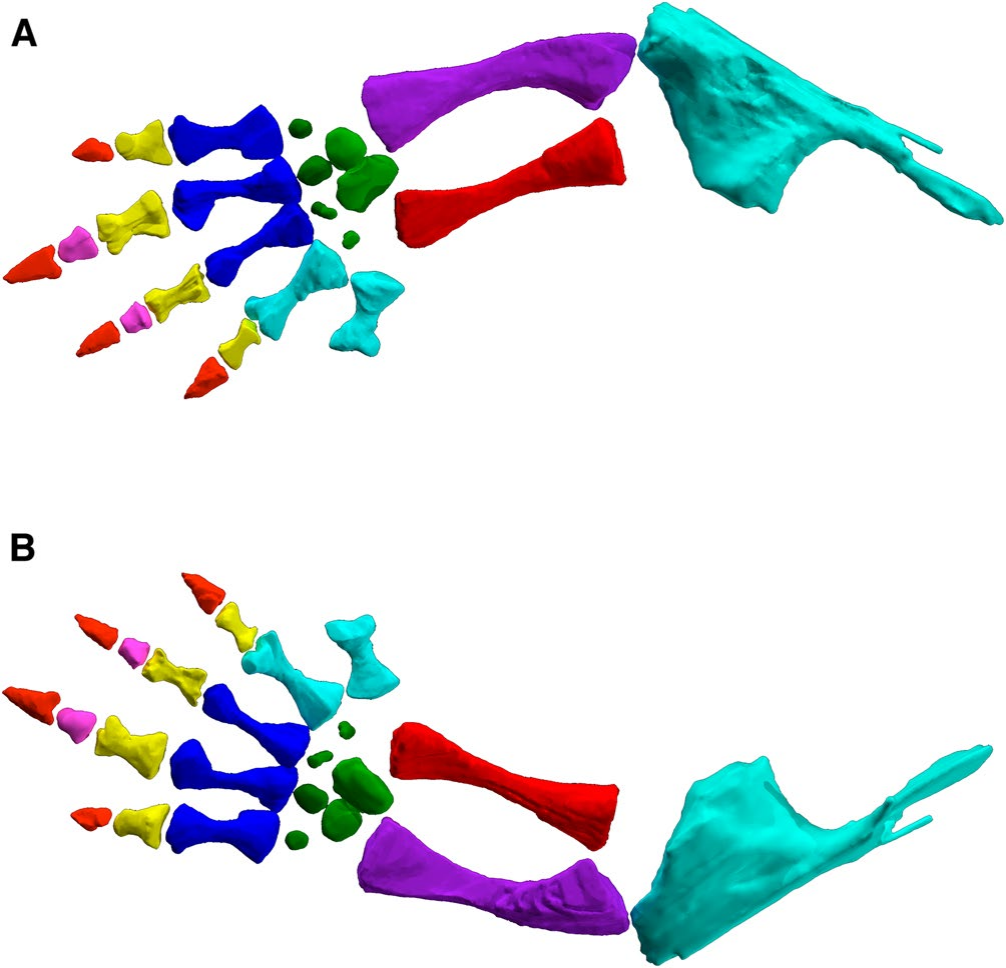
Reconstruction of the right forelimb of *Bromerpeton subcolossus*, MNG 16545, in a more natural position, in (**A**), dorsal, and (**B**), ventral views.

**Figure 7:**
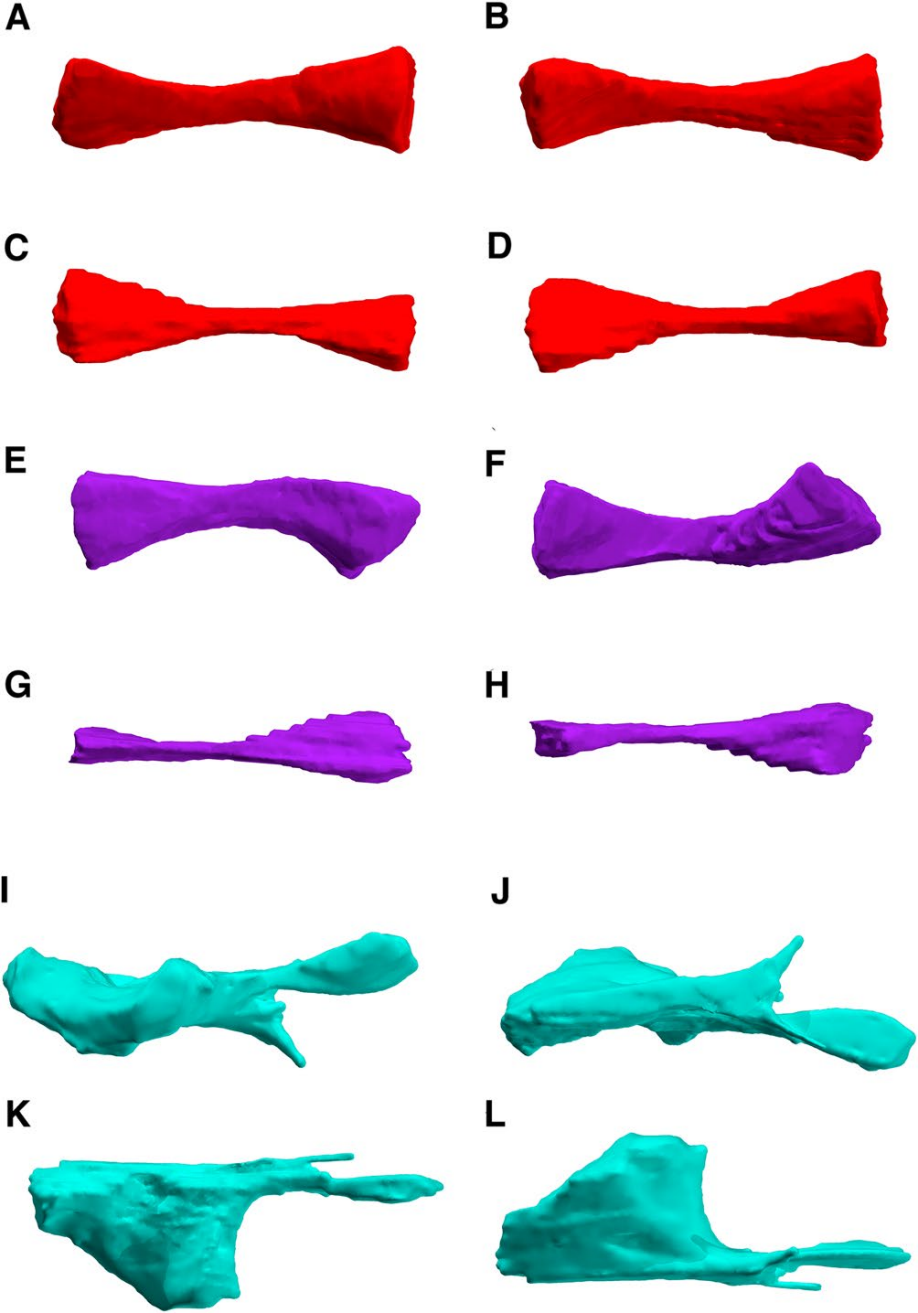
Reconstructions of the stylopodial and zeugopodial elements of the right forelimb of *Bromerpeton subcolossus*, MNG 16545. Right radius in (**A**), dorsal, (**B**), ventral, (**C**), lateral, and (**D**), medial views. Right ulna in (**E**), dorsal, (**F**), ventral, (**G**), lateral, and (**H**), medial views. Partial right humerus in (**I**), dorsal, (**J**), ventral, (**K**), lateral, and (**L**), medial views.

**Figure 8:**
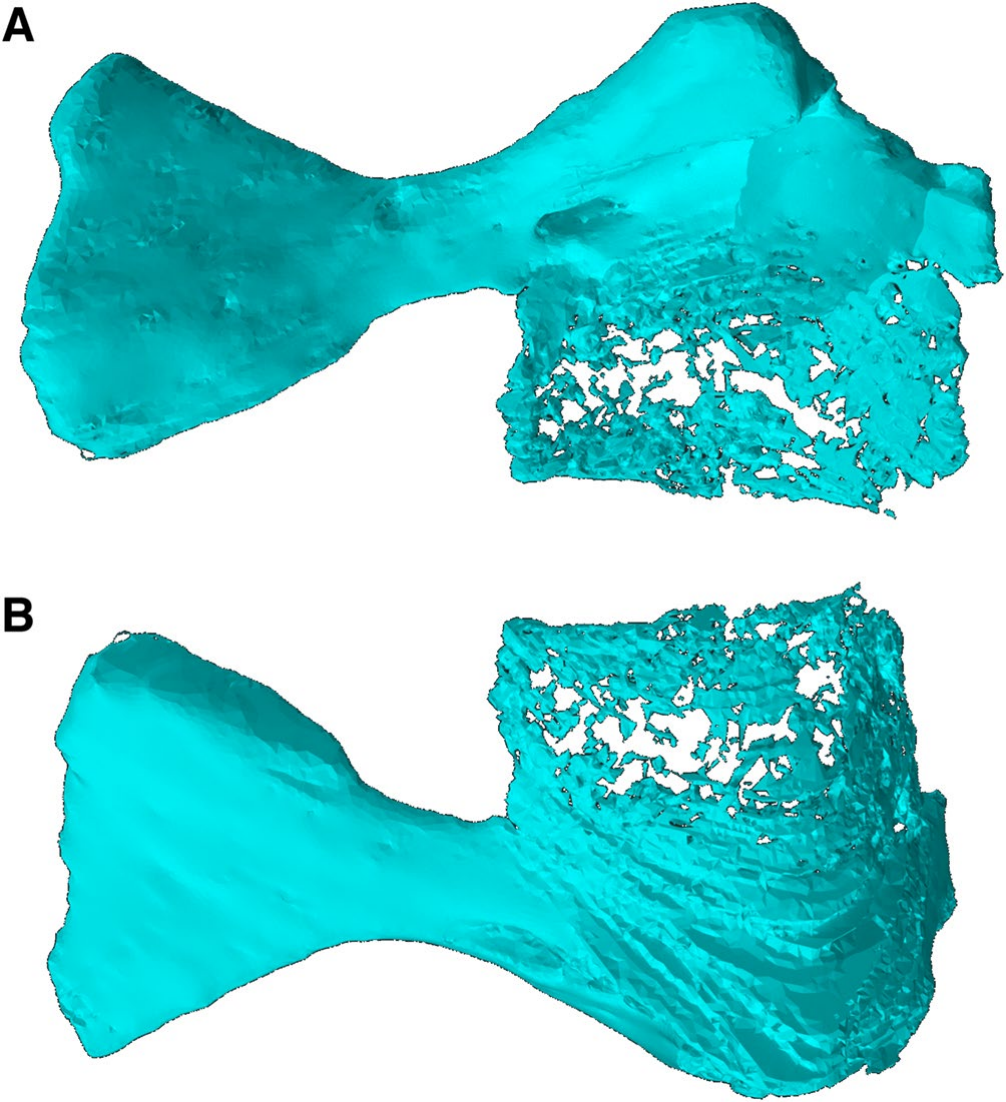
Reconstruction of the partial left humerus of *Bromerpeton subcolossus*, MNG 16545, in (**A**), dorsal, and (**B**), ventral views.

Only the forelimb elements are preserved, most notably the largely complete and articulated right forelimb (Figs. 1, 5, 6, 7) and the left humerus (Fig. 8). No vertebrae were identified, though there are some unidentifiable bone fragments scattered in the area where they would be expected. Measurements of elements of the forelimb are in Table 1.

**Table 1:** Forelimb measurements of the holotype of *Bromerpeton subcolossus*, MNG 16545.

	Humerus (mm)	Radius (mm)	Ulna (mm)
Length	18	9	10
Width (at widest point)	6	3	3

Both humeri are incomplete, with the right missing most of its proximal end, though its overall shape is recorded as a cast of its impression (Figs. 1, 5, 7), and the left having a damaged distal end (Figs. 1, 8). The humerus is robust and slightly longer than the radius and ulna, however the proximal and distal ends are much wider, being expanded into broad flat surfaces. The proximal end of the humerus also possesses a small deltopectoral crest. The two broad ends are separated by a short shaft, which contributes to less than a third of the length of the element. The humerus exhibits torsion of the shaft, with the distal end rotated almost 90 degrees relative to that of the proximal end. The degree of torsion is typical for ‘microsaurs’ and similar to that observed in various recumbirostrans for which the humerus is preserved, such as *Batropetes*^21^ and *Diabloroter*^29^. The distal end of the right humerus is closely associated with its respective radius and ulna. The left humerus possesses an oval shaped entepicondylar foramen on the distal end of the shaft that is not visible externally on the specimen but is clearly identifiable in the CT reconstruction of the element (Fig. 8).

The slender right radius and ulna are complete (Figs. 1, 5, 7), with the ulna being slightly longer. The radius has a straight shaft, whereas that of the ulna is bowed medially. The zeugopodial elements are roughly two-thirds the length of the humerus, and they do not differ dramatically from those of other recumbirostrans, though those in *Batropetes* appear to be slightly more robust^21^.

The right manus is nearly complete (Figs. 1, 5, 6), though a portion is not visible externally and was visualized using computed tomography (Figs. 5, 6). Six of the carpal elements are preserved with only two being exposed, which we identify as two of the proximal carpals. Their position and shape suggest that they are possibly the ulnare (lateralmost element) and the intermedium (medialmost element). The remaining four carpals visible in the CT scan are all likely distal carpals based on their position. The small size of these distal carpals is likely because we are seeing only a small, ossified portion of them; the remainder would have been cartilaginous. Portions of four digits of the right manus are visible externally, with one of the proximal digits being overlain and obscured by the more distal ones. The CT scan reveals the full extent of this obscured digit, and interestingly shows that five metacarpals are present, indicating that the manus had five digits rather than the expected four. Currently there are no known recumbirostrans with five manual digits, although the manus is unknown in most of them. The obscured metacarpal represents digit I, but unfortunately no associated phalanges are preserved. Thus, from what is preserved the phalangeal formula of the manus is ?, 2, 3, 3, 2. In the few recumbirostrans in which the manus is known they have either four digits^21,38^ or three digits^23^, making the presence of five in *Bromerpeton* unique among known recumbirostrans, which could be interpreted as a primitive state for the clade. The metacarpals and phalanges of the manus all appear to be quite robust, with the first metacarpal being shorter and broader than the others. The proximal phalanx of each digit is roughly half the length of its respective metacarpal and quite broad. The third and fourth digits have an additional smaller, penultimate phalanx, about half the length of the proximal phalanx, but remain quite robust. The unguals are broad, shovel-shaped, pointed, and slightly recurved, similar to *Batropetes*^21^.

## Results

**Figure 9:**
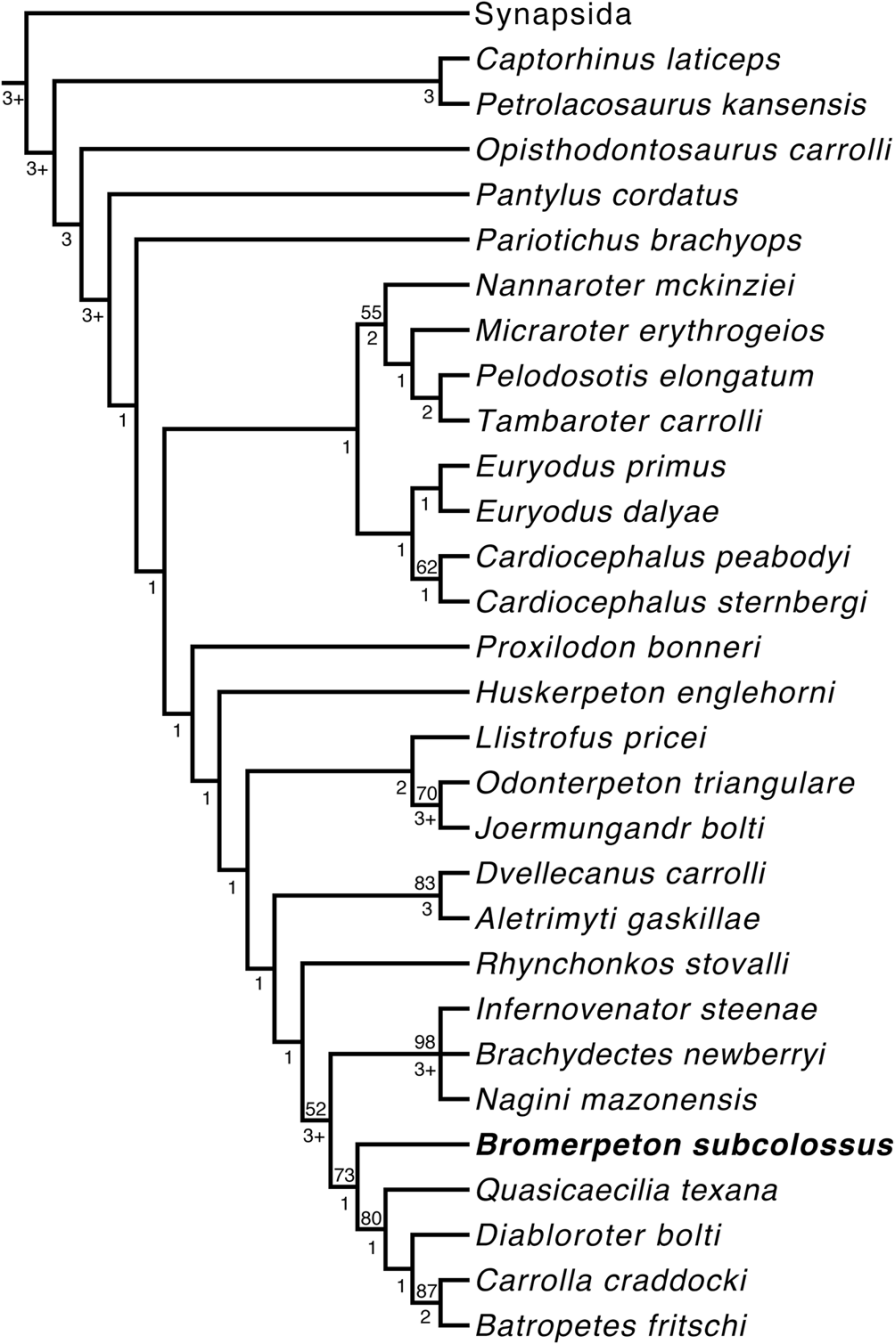
Consensus tree of the 18 most parsimonious trees obtained from the phylogenetic analysis. *Bromerpeton subcolossus* is recovered as the sister taxon to all other brachystelechids in both consensus trees. Tree length = 1782, Consistency index (CI) = 0.264, Rescaled CI = 0.173, Retention index = 0.654. Bootstrap support values above 50% are found above nodes and Bremer support values are found below nodes. Note that for clarity this tree has collapsed the synapsids included in the analysis into the clade Synapsida and anamniotes are not shown (complete tree can be found in Fig. S1). Both the strict consensus and 50% majority rule consensus produced identical topologies for this portion of the tree.

A phylogenetic analysis recovers *Bromerpeton subcolossus* as the basalmost member of the recumbirostran clade Brachystelechidae (Fig. 9; S1). This position is recovered in both the strict consensus tree and the majority rule consensus tree, both being identical regarding the interrelationships of the amniotes included in the analysis. Specifically, it is recovered as the sister taxon to a clade containing all other brachystelechids, the topology of which is identical to that recovered in another recent study of recumbirostrans^23^ with *Carrolla craddocki* and *Batropetes fritschi* as sister taxa, *Diabloroter bolti* as the sister taxon to this clade, and lastly *Quasiceacilia texana* as the most basal member.

The other recumbirostran from the Tambach Formation added to the matrix, *Tambaroter carrolli*, is recovered within the recumbirostran clade Ostodolepidae, as the sister taxon of *Pelodosotis elongatum.* This further supports the assignment of *Tambaroter* to Ostodolepidae that was made by Henrici et al.^19^.

## Discussion

### Phylogenetic position of Bromerpeton subcolossus

That *Bromerpeton* is recovered as a brachystelechid, a clade of miniaturized recumbirostran ‘microsaurs’^22^, is an interesting result, as it shares many similarities with known brachystelechids, such as smaller size, prominently wide parietals, and robust limbs, but also exhibits some key differences from other members of the clade. Most members of Brachystelechidae have multicuspid marginal teeth, although this is not the case for *Diabloroter bolti*^29^ or *Bromerpeton*. Both taxa have small simple monocuspid teeth, which is likely the plesiomorphic condition for the clade^29^ and supports the basal position of *Bromerpeton* as the sister taxon to all other brachystelechids.

An important difference of *Bromerpeton* from the other brachystelechids is its lack of a frontal contribution to the orbit, due to the prefrontal and postfrontal contact blocking the frontal from extending to the orbit; all other brachystelechids exhibit the orbital margin contribution of the frontal^21,28,29,32,39^. This feature in *Bromerpeton* could also be attributed to it being the most basal member of the clade and therefore a plesiomorphic condition of brachystelechids. Other than the brachystelechids and the hapsidopareiid *Llistrofus*^40,41^, all recumbirostrans lack a frontal contribution to the orbit, which also suggests it is the plesiomorphic condition for the entire clade.

The lack of a frontal contribution to the orbit in *Bromerpeton* could be related to its larger size when compared to other brachystelechids. *Bromerpeton* has a skull length of about 20 mm, whereas other brachystelechids have smaller skulls: *Quasicaecilia* and *Carrolla* have skull lengths of approximately 15 mm^28,32^, *Batropetes* has a skull length of approximately 10 mm^21^, and *Diabloroter* has a skull length of approximately 9.2 mm^29^. With decreasing body size of brachystelechids their relative orbit size increases, and they likely lost the contact between the prefrontal and postfrontal and gained a frontal contribution to the orbit. This evolutionary pattern parallels what is observed in amphibamid temnospondyls^42^.

Lastly, perhaps the most unexpected difference of *Bromerpeton* from other recumbirostrans is that it possesses five metacarpals in the manus, indicating it would have had five manual digits. In many recumbirostrans the manus is not known or has been completely lost, as is the case in some molgophids^35,43^. When the manus is present and preserved it possesses either three or four digits^21–23,38^. Thus, *Bromerpeton* possessing five manual digits adds important data on trends toward digit reduction within the clade, further supporting the hypothesis of limb reduction in fossorial recumbirostrans^43^. In *Batropetes* the four digits of the manus have a phalangeal formula of 2–3-3–1, whereas *Bromerpeton* has a formula of ?-2–3-3–2. This suggests that the first digit is lost within Brachystelechidae, which would indicate postaxial polarity during development. This has also been observed in the pes of the molgophid recumbirostran *Nagini mazonensis*^43^. Postaxial polarity refers to the order of digit formation during development, in this case the last digit to form is digit I, a condition that is observed in living amniotes and frogs^44^. Furthermore, studies on extant tetrapods have demonstrated that evolutionary digit loss occurs in reverse order relative to digit formation, i.e. the last digit to form developmentally is the first one lost during evolutionary digit loss^45–47^. The presence of five digits in the manus could potentially represent the primitive state for the clade, which was subsequently lost among other recumbirostrans.

### The Bromacker ecosystem and the possible ecology of Bromerpeton

The Bromacker locality is interpreted as an internally drained basin located far inland in eastern Laurasia^4^. Furthermore, it represents the most fossiliferous and species rich lower Permian terrestrial locality located in Europe^18^. Inland lower Permian localities are extremely rare with there only being a few others currently known worldwide^5,48^. The number of taxa known from Bromacker, combined with the abundance and completeness of fossil material, makes it one of the richest fossil assemblages currently known for studying the palaeoecology of a lower Permian ecosystem.

The best known and most complete taxa from the locality are generally medium to large-sized vertebrates, such as the seymouriamorph *Seymouria sanjuanensis*^1^, the diadectids *Diadectes absitus*^10^ and *Orobates pabsti*^2^, the varanopid *Tambacarnifex unguifalcatus*^17^, and the synapsids *Dimetrodon teutonis*^16^ and *Martensius bromackerensis*^18^. However, there are also various smaller tetrapod taxa that have been described from the Bromacker locality, including the basal captorhinomorph *Thuringothyris mahlendorffae*^15^, the bolosaurid *Eudibamus cursoris*^14^, the amphibamid *Georgenthalia clavinasica*^13^, and the trematopids *Tambachia trogallas* and *Rotaryus gothae*^11,12^. Generally, these smaller taxa are not as common at the locality, but still offer highly relevant information regarding the ecosystem composition and structure that would have been present at Bromacker. *Bromerpeton subcolossus* is no exception in that it gives us important information about the small predators that would have been living alongside the herbivorous diadectids that dominated the Bromacker assemblage. With there being many ‘microsaurs’ known from the early Permian of Germany^19–22^, it is likely they were a common faunal component of early Permian ecosystems in this region of Laurasia.

As stated earlier *Bromerpeton* differs from the other members of the recumbirostran clade Brachystelechidae (with the exception of *Diabloroter*) with its simple monocuspid marginal dentition. The small pointed peg-like teeth of *Bromerpeton* are reminiscent of the type exhibited by many early reptiles, which is usually ascribed to a predatory lifestyle with arthropods or smaller tetrapods being the main prey item^49,50^. This is also the lifestyle that has been attributed to the brachystelechid *Diabloroter* on the basis of its similar dentition^29^. Thus, it is likely that *Bromerpeton* would have had an insectivorous diet, preying on the arthropods and/or myriapods that are known to be present at Bromacker^4,51^. This is a dietary guild it would have shared with other taxa at Bromacker that also exhibit a similar size and dentition, such as *Thuringothyris*^15^, *Georgenthalia*^13^, *Rotaryus*^12^, and likely the juveniles of *Martensius*^18^ and *Seymouria*^1^. This lifestyle as a small insectivorous predator would have also likely been shared with its recumbirostran relative, *Tambaroter carrolli,* also known from the Tambach Formation^19^.

*Bromerpeton* also exhibits several skeletal features that suggest it could have been fossorial. The skull possesses a characteristic recumbent snout and would have been roughly triangular in shape due to its broad parietals, two features that are often considered to represent possible fossorial adaptations in recumbirostrans^28,31,52^. Specifically, based on preserved elements the recumbent snout appears to be of the round-headed ecomorph, similar to what is observed in *Carrolla*^35^. Increased sutural complexity is another feature that has been attributed to fossoriality in fossil tetrapods^53^. The skull of *Bromerpeton* may have had highly interdigitated sutures between the nasals and frontals, but unfortunately due to the nasals being very fragmentary, this is only visible between a fragment of the left nasal (Fig. 1) and its associated frontal. Thus, it is unknown whether this type of interdigitation would have continued for the entire nasal-frontal contact. The forelimb of *Bromerpeton* also possesses several features that suggest fossoriality, specifically its pointed shovel shaped unguals and overall broad manus (Figs. 5,6). *Batropetes*, a close relative of *Bromerpeton*, has been attributed a scratch-digging mode of fossoriality^54^, it is possible that *Bromerpeton* could have also exhibited a similar type of digging. Furthermore, the plesiomorphic condition of five manual digits in *Bromerpeton* and the subsequent reduction of manual digits in other brachystelechids is interesting with regards to the evolution of fossoriality within the clade. The digit reduction within the clade could have allowed for an increase in rigidity of the forelimb to better accommodate a fossorial lifestyle, as is observed in some extant fossorial tetrapods^55,56^.

## Conclusions

We describe a new ‘microsaur’ taxon, *Bromerpeton subcolossus* gen. et sp. nov., from the lower Permian Bromacker locality of Germany, the second microsaur to be described from the Tambach Formation. Phylogenetic analysis indicates that it represents the basalmost member of the recumbirostran clade Brachystelechidae. *Bromerpeton* is distinguished from other brachystelechids by its monocuspid dentition, presence of 13 maxillary teeth, lack of a frontal contribution to the orbital margin, postorbitals not contributing substantially to the postorbital region of the skull, and the presence of five digits in the manus, which all represent plesiomorphic characters that support its basal position within the clade. *Bromerpeton* was likely a small insectivorous predator within the Bromacker faunal assemblage, in contrast to the large herbivores that dominated the assemblage. Characteristics of its skull and forelimb also suggest that it would have been capable of fossorial behaviour. Most notably, the presence of five manual digits, a feature unique among recumbirostrans, provides important data for understanding limb reduction and limb loss in fossorially adapted recumbirostrans. Overall, this new taxon further adds to the extensive species richness of the unique early Permian Bromacker fauna, as well as to our knowledge of the palaeobiology and macroevolution of recumbirostran ‘microsaurs’ in general.

### Supplementary Information

Supplementary Figure S1.

Supplementary Information 2.

## Data Availability

The phylogenetic dataset used in this study can be found in the supplementary information. The raw CT data used can be accessed at MorphoSource, https://www.morphosource.org/projects/000449864.

## References

[1] Berman DS, Henrici AC, Sumida SS, Martens T (2000). Redescription of *Seymouria sanjuanensis* (Seymouriamorpha) from the Lower Permian of Germany based on complete, mature specimens with a discussion of paleoecology of the Bromacker locality assemblage. J. Vertebr. Paleontol..

[2] Berman DS, Henrici AC, Kissel RA, Sumida SS, Martens T (2004). A new diadectid (Diadectomorpha), *Orobates pabsti*, from the early Permian of central Germany. Bull. Carnegie Mus. Nat. Hist..

[3] Berman DS, Henrici AC, Sumida SS, Martens T (2023). Origin of the modern terrestrial vertebrate food chain. Ann. Carnegie Mus..

[4] Eberth DA, Berman DS, Sumida SS, Hopf H (2000). Lower Permian terrestrial paleoenvironments and vertebrate paleoecology of the Tambach Basin (Thuringia, Central Germany): The upland holy grail. PALAIOS.

[5] MacDougall MJ, Tabor NJ, Woodhead J, Daoust AR, Reisz RR (2017). The unique preservational environment of the Early Permian (Cisuralian) fossiliferous cave deposits of the Richards Spur locality, Oklahoma. Palaeogeogr. Palaeoclimatol. Palaeoecol..

[6] Schneider JW (2020). Late Paleozoic–early Mesozoic continental biostratigraphy—links to the standard global chronostratigraphic scale. Palaeoworld.

[7] Lützner H, Tichomirowa M, Käßner A, Gaupp R (2020). Latest Carboniferous to early Permian volcano-stratigraphic evolution in Central Europe: U-Pb CA–ID–TIMS ages of volcanic rocks in the Thuringian Forest Basin (Germany). Int. J. Earth Sci..

[8] Martens, T. *Taxonomie und Biostratigraphie der Conchostraken (Phyllopoda, Crustacea) aus dem terrestrischen Oberen Pennsylvanian und Cisuralian (unteres Perm) von Nord-Zentral Texas (USA): Taxonomy and biostratigraphy of conchostracans (Phyllopoda, Crustacea) from the terrestrial Upper Pennsylvanian and Cisuralian (lower Permian) of North-Central Texas (USA)* (Cuvillier, 2020).

[9] Menning M (2022). The Rotliegend in the stratigraphic table of Germany 2016 (STG 2016). Z. Dtsch. Ges. Für Geowiss..

[10] Berman DS, Sumida SS, Martens T (1998). *Diadectes* (Diadectomorpha: Diadectidae) from the Early Permian of central Germany, with description of a new species. Ann. Carnegie Mus. Pittsburgh.

[11] Sumida SS, Berman DS, Martens T (1998). A new trematopid amphibian from the Lower Permian of central Germany. Palaeontology.

[12] Berman DS, Henrici AC, Martens T, Sumida SS, Anderson JS (2011). *Rotaryus gothae*, a new trematopid (Temnospondyli: Dissorophoidea) from the Lower Permian of Central Germany. Ann. Carnegie Mus..

[13] Anderson JS, Henrici AC, Sumida SS, Martens T, Berman DS (2008). *Georgenthalia clavinasica*, a new genus and species of dissorophoid temnospondyl from the Early Permian of Germany, and the relationships of the family Amphibamidae. J. Vertebr. Paleontol..

[14] Berman DS (2000). Early Permian bipedal reptile. Science.

[15] Müller J, Berman DS, Henrici AC, Martens T, Sumida SS (2006). The basal reptile *Thuringothyris mahlendorffae* (Amniota: Eureptilia) from the Lower Permian of Germany. J. Paleontol..

[16] Berman DS, Reisz RR, Martens T, Henrici AC (2001). A new species of *Dimetrodon* (Synapsida: Sphenacodontidae) from the Lower Permian of Germany records first occurrence of genus outside of North America. Can. J. Earth Sci..

[17] Berman, D. S., Henrici, A. C., Sumida, S. S., Martens, T. & Pelletier, V. In *Early Evol. Hist. Synapsida* (eds. Kammerer, C. F. *et al*.) 69–86 (Springer Netherlands, 2014).

[18] Berman DS (2020). New primitive caseid (Synapsida, Caseasauria) from the Early Permian of Germany. Ann. Carnegie Mus..

[19] Henrici AC, Martens T, Berman DS, Sumida SS (2011). An ostodolepid ‘microsaur’ (Lepospondyli) from the Lower Permian Tambach Formation of central Germany. J. Vertebr. Paleontol..

[20] Glienke S (2012). A new “microsaur” (Amphibia; Lepospondyli) from the Rotliegend of the Saar-Palatinate region (Carboniferous/Permian transition; West Germany). Paläontol. Z..

[21] Glienke S (2015). Two new species of the genus *Batropetes* (Tetrapoda, Lepospondyli) from the Central European Rotliegend (basal Permian) in Germany. J. Vertebr. Paleontol..

[22] Carroll RL, Gaskill P (1978). The Order Microsauria.

[23] Mann A, Pardo JD, Sues H-D (2022). Osteology and phylogentic position of the diminuitive ‘microsaur’ *Odonterpeton triangulare* from the Pennsylvanian of Linton, Ohio, and major features of recumbirostran phylogeny. Zool. J. Linn. Soc..

[24] MacDougall MJ (2021). Revised description of the early Permian recumbirostran “microsaur” *Nannaroter mckinziei* based on new fossil material and computed tomographic data. Front. Ecol. Evol..

[25] Swofford DLP (2003). Phylogenetic Analysis Using Parsimony (*and Other Methods).

[26] Jaekel O (1909). Ūber die Klassen der Tetrapoden. Zool. Anz..

[27] Anderson, J. S. Incorporating ontogeny into the matrix: A phylogenetic evaluation of developmental evidence for the origins of modern amphibians. In *Major Transitions in Vertebrate Evolution* (eds. Anderson, J. S. & Sues, H.-D.) 182–227 (Indiana University Press, 2007).

[28] Pardo JD, Szostakiwskyj M, Anderson JS (2015). Cranial morphology of the brachystelechid ‘microsaur’ *Quasicaecilia texana* Carroll provides new insights into the diversity and evolution of braincase morphology in recumbirostran ‘microsaurs’. PLOS ONE.

[29] Mann A, Maddin HC (2019). *Diabloroter bolti*, a short-bodied recumbirostran ‘microsaur’ from the Francis Creek Shale, Mazon Creek, Illinois. Zool. J. Linn. Soc..

[30] Huttenlocker A, Pardo JD, Small BJ, Anderson JS (2013). Cranial morphology of recumbirostrans (Lepospondyli) from the Permian of Kansas and Nebraska, and early morphological evolution inferred by micro-computed tomography. J. Vertebr. Paleontol..

[31] Szostakiwskyj M, Pardo JD, Anderson JS (2015). Micro-CT study of *Rhynchonkos stovalli* (Lepospondyli, Recumbirostra), with description of two new genera. PLOS ONE.

[32] Maddin HC, Olori JC, Anderson JS (2011). A redescription of *Carrolla craddocki* (Lepospondyli: Brachystelechidae) based on high-resolution CT, and the impacts of miniaturization and fossoriality on morphology. J. Morphol..

[33] Gee BM, Bevitt JJ, Reisz RR (2020). Computed tomographic analysis of the cranium of the early Permian recumbirostran ‘microsaur’ *Euryodus dalyae* reveals new details of the braincase and mandible. Pap. Palaeontol..

[34] Mann A (2020). Reassessment of historic ‘microsaurs’ from Joggins, nova Scotia, reveals hidden diversity in the earliest amniote ecosystem. Pap. Palaeontol..

[35] Mann A, Pardo JD, Maddin HC (2019). *Infernovenator steenae*, a new serpentine recumbirostran from the ‘Mazon Creek’ Lagerstätte further clarifies lysorophian origins. Zool. J. Linn. Soc..

[36] MacDougall MJ, LeBlanc ARH, Reisz RR (2014). Plicidentine in the Early Permian parareptile *Colobomycter pholeter*, and its phylogenetic and functional significance among coeval members of the clade. PLoS ONE.

[37] Mann A, Calthorpe AS, Maddin HC (2021). *Joermungandr bolti*, an exceptionally preserved ‘microsaur’ from the Mazon Creek Lagerstätte reveals patterns of integumentary evolution in Recumbirostra. R. Soc. Open Sci..

[38] Wellstead CF (1991). Taxonomic revision of the Lysorophia, Permo-Carboniferous Lepospondyl amphibians. Bull. Am. Mus. Nat. Hist..

[39] Carroll RL (1990). A tiny microsaur from the lower Permian of Texas: Size constraints in palaeozoic tetrapods. Palaeontology.

[40] Bolt JR, Rieppel O (2009). The holotype skull of *Llistrofus pricei* Carroll and Gaskill, 1978 (Microsauria: Hapsidopareiontidae). J. Paleontol..

[41] Gee BM, Bevitt JJ, Garbe U, Reisz RR (2019). New material of the ‘microsaur’ *Llistrofus* from the cave deposits of Richards Spur, Oklahoma and the paleoecology of the Hapsidopareiidae. PeerJ.

[42] Fröbisch NB, Schoch RR (2009). Testing the impact of miniaturization on phylogeny: Paleozoic dissorophoid amphibians. Syst. Biol..

[43] Mann A, Pardo JD, Maddin HC (2022). Snake-like limb loss in a Carboniferous amniote. Nat. Ecol. Evol..

[44] Fröbisch NB (2008). Ossification patterns in the tetrapod limb—conservation and divergence from morphogenetic events. Biol. Rev..

[45] Alberch P, Gale EA (1983). Size dependence during the development of the amphibian foot. Colchicine-induced digital loss and reduction. J. Embryol. Exp. Morphol..

[46] Shubin NH, Alberch P (1986). A morphogenetic approach to the origin and basic organisation of the tetrapod limb. Evol. Biol..

[47] Stopper GF, Wagner PG (2007). Inhibition of Sonic hedgehog signaling leads to posterior digit loss in *Ambystoma mexicanum*: Parallels to natural digit reduction in urodeles. Dev. Dyn..

[48] Olson EC (1967). Early Permian vertebrates of Oklahoma. Okla. Geol. Surv..

[49] Modesto SP, Scott DM, Reisz RR (2009). Arthropod remains in the oral cavities of fossil reptiles support inference of early insectivory. Biol. Lett..

[50] MacDougall MJ, Modesto SP, Reisz RR (2016). A new reptile from the Richards Spur locality, Oklahoma, USA, and patterns of Early Permian parareptile diversification. J. Vertebr. Paleontol..

[51] Martens T, Schneider J, Walter H (1981). Zur Paläeontologie und Genese fossilfuehrender Rotsedimende der Tambacher Sandstein, Oberrotliegendes, Thüringer Wald (DDR). Freib. Forschungsh. Reihe C.

[52] Anderson JS, Scott D, Reisz RR (2009). Nannaroter mckinziei, a new ostodolepid ‘microsaur’ (Tetrapoda, Lepospondyli, Recumbirostra) from the Early Permian of Richards Spur (Ft. Sill), Oklahoma. J. Vertebr. Paleontol..

[53] Kammerer CF (2021). Elevated cranial sutural complexity in burrowing dicynodonts. Front. Ecol. Evol..

[54] Jansen M, Marjanović M (2022). The scratch-digging lifestyle of the Permian ‘microsaur’ *Batropetes* Carroll & Gaskill, 1971 as a model for the exaptative origin of jumping locomotion in frogs. Comp. Rend. Palevol.

[55] Heideman NJL, Mulcahy DG, Sites JW, Hendricks MGJ, Daniels SR (2011). Cryptic diversity and morphological convergence in threatened species of fossorial skinks in the genus *Scelotes* (Squamata: Scincidae) from the Western Cape Coast of South Africa: Implications for species boundaries, digit reduction and conservation. Mol. Phylogenet. Evol..

[56] Hildebrand M (2013). Digging of Quadrupeds in Functional Vertebrate Morphology.

