# Author Correction: A new recumbirostran ‘microsaur’ from the lower Permian Bromacker locality, Thuringia, Germany, and its fossorial adaptations

**DOI:** 10.1038/s41598-024-59039-x

**Published:** 2024-04-25

**Authors:** Mark J. MacDougall, Andréas Jannel, Amy C. Henrici, David S. Berman, Stuart S. Sumida, Thomas Martens, Nadia B. Fröbisch, Jörg Fröbisch

**Affiliations:** 1 Museum für Naturkunde Leibniz-Institut für Evolutions- und Biodiversitätsforschung, Invalidenstraße 43, 10115 Berlin, Germany; 2 Carnegie Museum of Natural History, Pittsburgh, PA USA; 3 California State University, San Bernardino, CA USA; 4 99869 Drei Gleichen, Germany; 5 Humboldt-Universität zu Berlin, Invalidenstraße 42, 10115 Berlin, Germany

Correction to: *Scientific Reports* 10.1038/s41598-023-46581-3, published online 20 February 2024

The original version of this Article did not include evidence of registration in ZooBank within the work itself^1^, as is required by Article 8.5.3 of the International Code of Zoological Nomenclature^2^. Therefore, the new genus name, *Bromerpeton*, and species name, *Bromerpeton subcolossus*, are not available from that article. This issue is corrected here and the new taxon name is now available.

## Systematic Palaeontology

TETRAPODA Jaekel, 1909^3^

RECUMBIROSTRA Anderson, 2007^4^

BRACHYSTELECHIDAE Carroll and Gaskill, 1978^5^

*BROMERPETON SUBCOLOSSUS* gen. et sp. nov.

(Figs. 1–3 in MacDougall et al. (2024)^1^)

[urn:lsid:zoobank.org:act:78B072B4-27B3-42B3-8E67-7D349A4F719A (genus)]

[urn:lsid:zoobank.org:act:769BA815-ADFA-4C05-9769-F044C37392AA (species)]

*Holotype*: MNG 16545, a partial skull and mandible with left humerus and largely complete right forelimb.

*Diagnosis:* Brachystelechid recumbirostran diagnosed by the following characters: 13 maxillary teeth, frontals do not contribute to the orbital margin, narrow postorbitals do not contribute substantially to the postorbital region of the skull, and presence of five manual digits. In all other brachystelechids the prefrontal and postfrontal do not meet, which allows the frontal to contribute to the dorsal edge of the orbital margin. Shares with *Diabloroter*, but differs from *Carrolla* and *Batropetes* in exhibiting a homodont dentition of small monocuspid teeth.

*Etymology*: Genus name derives from Bromacker (which translates to Brom’s Field in English) the name of the locality that the specimen was discovered at, and *erpeton* is a common epithet for small early tetrapods, meaning creeper in Greek. Species name derives from the Latin words for below and colossus, referring to the small size of this species relative to the abundant and much larger vertebrates of the fauna.

*Locality and horizon:* Bromacker locality, quarry near the town of Tambach-Dietharz, Thuringia, Germany. Located in the Upper Beds of the Lower Permian Tambach Formation^6^, Artinskian in age based on biostratigraphy^7^, but potentially could be as old as early Sakmarian/late Asselian^8–10^.

## Nomenclatural acts

The electronic version of this article conforms to the requirements of the International Code of Zoological Nomenclature (ICZN), and the new names contained herein are available under that Code from the electronic version of this article. This published work and the nomenclatural acts it contains have been registered in ZooBank, the proposed online registration system for the ICZN. The ZooBank Life Science Identifiers (LSIDs) can be resolved and the associated information viewed through any standard web browser by appending the LSID to the prefix http://zoobank.org/. The LSIDs for this publication are: urn:lsid:zoobank.org:pub:09C76579-1F21-4E7A-8C85-CF7FFE6FF6A7 (article); urn:lsid:zoobank.org:act:78B072B4-27B3-42B3-8E67-7D349A4F719A (genus); urn:lsid:zoobank.org:act:769BA815-ADFA-4C05-9769-F044C37392AA (species).

The original Article has been corrected.
