# Supplementary material for: A new recumbirostran ‘microsaur’ from the lower Permian Bromacker locality, Thuringia, Germany, and its fossorial adaptations
Source: Sci Rep. 2024 Feb 20;14:4200. doi: 10.1038/s41598-023-46581-3 (PMC10879142; doi:10.1038/s41598-023-46581-3)
Supplement: Supplementary file 1 — Supplementary Figure S1. [file 41598_2023_46581_MOESM1_ESM.docx]

**
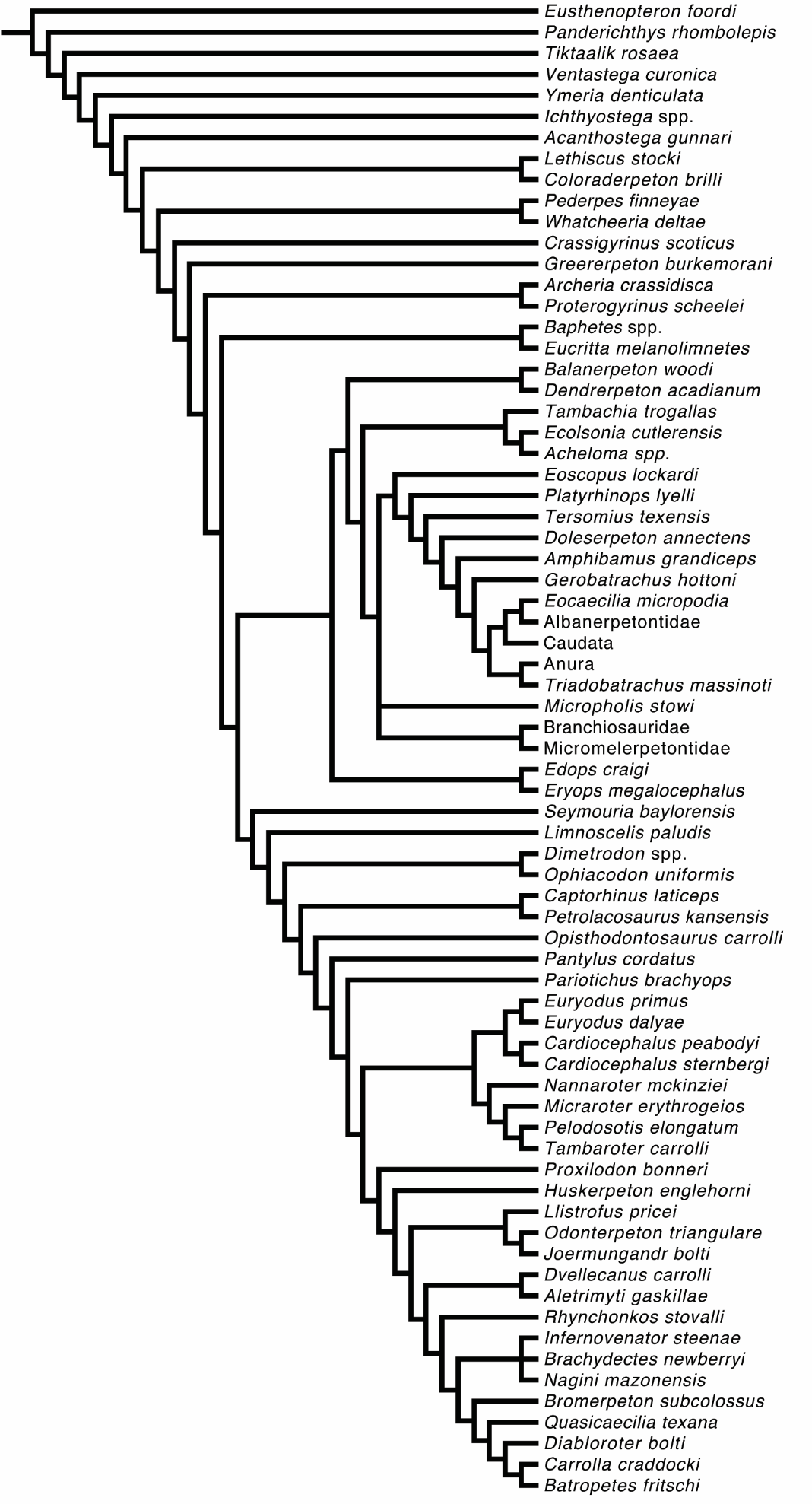
**

Figure S1. Strict consensus of the 18 most parsimonious trees obtained from the phylogenetic analysis.
